# Evaluation of the antioxidant and endothelial protective effects of *Lysimachia christinae* Hance (*Jin Qian Cao*) extract fractions

**DOI:** 10.1186/s12906-018-2157-1

**Published:** 2018-04-10

**Authors:** Ning-hua Wu, Zhi-qiang Ke, Shan Wu, Xiao-song Yang, Qing-jie Chen, Sheng-tang Huang, Chao Liu

**Affiliations:** 1 Hubei Key Laboratory of Cardiovascular, Cerebrovascular, and Metabolic Disorders, Hubei University of Science and Technology, Xianning, China; 2 Xianning Maternal and Child Health Care Hospital, Xianning, China

**Keywords:** *Lysimachia christinae* Hance, Radical-scavenging, Antioxidant, HUVECs, Flavonoids

## Abstract

**Background:**

*Lysimachia christinae* Hance is a traditional Chinese medicine with diuretic, detumescent, and detoxifying effects. Our aimed to optimize the extraction protocol to maximize the yield of flavonoids from *Lysimachia christinae* Hance, and evaluate the pharmacological activities of four fractions, namely, petroleum ether (PE), ethyl acetate (EA), n-butanol (NB), and aqueous (AQ) fractions, of the ethanolic extract of *Lysimachia christinae* Hance.

**Methods:**

The flavonoid monomers in the crude extract were characterized via high performance liquid chromatography (HPLC), were used as markers for extract quality control and standardization. The total flavonoid, total phenolic, and total polysaccharide contents of each fraction were determined by spectrophotometry. Further, the in vitro free radical (diphenylpicrylhydrazyl, 2,2′-azino-bis(3-ethylbenzothiazoline-6-sulphonic acid), superoxide, and hydroxyl radicals) scavenging activities, and antioxidant capacity in endothelial cells were evaluated for each fraction.

**Results:**

After optimizing the extraction protocol to maximize the total flavonoid yield from *L. christinae* Hance, the NB fractions had the highest total flavonoid (39.4 ± 4.55 mg RE/g), total phenolic (41.1 ± 3.07 mg GAE/g) and total polysaccharide (168.1 ± 7.07 mg GE/g); In addition, the NB fraction of the ethanolic extract of *L. christinae* Hance reveal the strongest radical-scavenging activity, antioxidant activity and protective effects against H_2_O_2_-induced injury in HUVECs.

**Conclusions:**

Among the four fractions of *L. christinae* Hance, the NB fraction showed the most potent antioxidant and endothelial protective effects, which may be attributed to its high flavonoid, phenolic contents and optimal portfolio of different active ingredients of NB fractions of the ethanolic extract of *L. christinae* Hance. This study might improve our understanding of the pharmacological activities of *L. christinae* Hance, thereby facilitating its use in disease prevention and treatment.

## Background

*Lysimachia christinae* Hance (*Jin Qian Cao*), which belongs to the family *Primulaceae*, was recorded in the 2010 edition of the Chinese Pharmacopoeia as an herb that possesses diuretic, detumescent, and detoxifying effects. It has been widely used for treating hepatobiliary lithiasis, urolithiasis, heat stranguria, nephritis edema, damp jaundice, and carbuncle in traditional Chinese medicine (TCM) [1, 2] and is therefore being increasingly investigated for its therapeutic potential [3, 4]. Although chemical constituent analyses indicate that *L. christinae* Hance contains several bioactive constituents, flavonoids, phenols, polysaccharides, triterpenoid saponins, volatile oils, organic acids, and quinones, the identity of these constituents and their pharmacological activities remain poorly researched.

Reactive oxygen species (ROS), which include free radicals, such as superoxide anion (O_2_^•-^) and hydroperoxyl radical (^•^OH), and non-free radicals, such as hydrogen peroxide (H_2_O_2_), act as signaling molecules and are constantly produced in living cells. Although they are efficiently eliminated by antioxidant defense systems under physiological conditions, an imbalance between ROS production and elimination leads to oxidative stress, which can damage biomolecules such as DNA, proteins, and lipids and greatly contribute to increased cardiovascular risk [5]. Such undesirable outcomes of oxidative stress can be effectively counteracted only by boosting endogenous antioxidant defenses or via supplementation with exogenous antioxidants [6].

The potential of naturally occurring plant flavonoids as exogenous (i.e., dietary) antioxidants and their application in the prevention and/or treatment of cardiovascular diseases over the last decades has been well-documented [7–9]. The hydroalcoholic extract of *Lysimachia clethroides*, another plant of the genus *Lysimachia*, has been reported to protect vascular function via an endothelium-dependent mechanism [10]. Further, our pilot data suggested that *L. christinae* Hance extract could inhibit ROS foramtion in HUVECs. Therefore, the present study was designed to evaluate the antioxidant potential and endothelial protective effects of *L. christinae* Hance, which was extracted with ethanol and separated into four fractions using solvents of different polarities.

## Methods

### Chemicals and reagents

*L. christinae* Hance herbs (Harvested in October 2014 from Sichuan, China, subsequently dry and stored under the condition of dark) were purchased from Kang Jin Pharmaceutical (Xianning, China). Butylated hydroxytoluene (BHT), H_2_O_2_, and acetylcholine (ACh) were purchased from Sigma-Aldrich (St Louis, MO, USA). Ascorbic acid (Vitamin C) and Trolox were purchased from Richu Biosciences (Shanghai, China). Diphenylpicrylhydrazyl (DPPH) and 2,2′-azino-bis(3-ethylbenzothiazoline-6 -sulphonic acid) (ABTS) were obtained from Aladdin Reagent (Shanghai, China). Rutin was purchased from Sangon Biotech (Shanghai, China). Dihydroethidium (DHE) was obtained from Thermo Fisher Scientific (MA, USA). 4′,6-diamidino-2-phenylindole (DAPI) was obtained from Calbiochem (Madrid, Spain). All other reagents used were of analytical grade. Double distilled water was used throughout the experiments.

### Preparation of *L. christinae* Hance extract

The crude extract of *L. christinae* Hance was prepared in the Phytochemistry Laboratory, Department of Materia Medica, Hubei University of Science and Technology (Xianning, China). Dried herb powder (50 g) was first extracted using 75% ethanol at 60 °C for 30 min. This extraction protocol was optimized to maximize the total flavonoid yield and standardized via identification and quantification of certain markers (flavonoid monomers) in the extract by ultraviolet-high performance liquid chromatography (UV-HPLC). The crude ethanolic extract was then fractionated into the petroleum ether (PE), ethyl acetate (EA), n-butanol (NB), and aqueous (AQ) fractions. The solvents were evaporated, and the residues were dried under vacuum. Finally, the four fractions of different polarities were stored at 4 °C in the dark until use.

### Qualitative and quantitative analysis of flavonoid monomers in the ethanolic extract

Certain flavonoid monomers in the crude ethanolic extract, designated as markers for quality control and standardization, were characterized by UV-HPLC. The analyses were performed on a LC-20 AD system (Shimadzu, Kyoto, Japan) equipped with an online degasser and a UV detector (G1314B, Agilent Technologies, CA, USA), using a C18 column (ZORBAX SB-C18, 4.6 × 150 mm, 5 μm, Agilent Technologies) maintained at 30 °C and a mobile phase consisting of methanol, acetonitrile, and 1% phosphoric acid in water, at a flow rate of 0.8 mL/min. The eluent was monitored at 370 nm. The most optimal chromatographic conditions were adopted to achieve the best peak resolution and shortest analysis times. The data were analyzed using LabSolutions software (version 5.51, Shimadzu).

### Estimation of the total flavonoid, total phenolic, and total polysaccharide contents of the extract fractions

The total flavonoid and phenolic contents and total polysaccharide content of each fraction were determined spectrophotometrically by the protocol of Asaduzzaman [11, 12], and phenol-sulphuric acid method, respectively. All experiments were performed in triplicate. The total flavonoid, total phenolic, and total polysaccharide contents were expressed as milligrams of rutin equivalents (RE), gallic acid equivalents (GAE), and glucose equivalents (GE), respectively, per gram of extract.

### Determination of the in vitro radical-scavenging activities of the extract fractions

The in vitro DPPH, ABTS, superoxide anion, and hydroxyl radical-scavenging activities of the four fractions were assayed as previously described [4, 13–15].

### Cell culture and treatment

Human umbilical vein endothelial cells (HUVECs) and cell culture medium (ECM containing 5% fetal bovine serum (FBS), 1% ECGs, 100 U/mL penicillin, and 100 μg/mL streptomycin) were purchased from ScienCell Research Laboratories (San Diego, CA, USA). Cells between passages 2 and 5 were maintained at 37 °C in a humidified atmosphere of 5% CO_2_ and 95% air. Confluent cells were treated with the same dose (6.7 μg/ mL) of each fraction, 2 h prior to exposure to 50 μM H_2_O_2_ for 5 h. Thus, using an oxidative stress model based on H_2_O_2_-induced injury, we compared the antioxidant activity of the four extract fractions.

### Determination of cell viability

The cell viability was measured in 96-well plates via the non-radioactive cell counting kit-8 (CCK-8) assay (Dojindo Molecular Technologies, Kumamoto, Japan), according to the manufacturer’s instructions.

### Determination of the antioxidant activity of the extract fractions

The catalase (CAT) activity,the levels of glutathione (GSH), malondialdehyde (MDA) and Nitric Oxide (NO), and the lipid peroxidation marker, in HUVECs were measured by colorimetry using commercially available biochemical kits (Nanjing Jiancheng Bioengineering Research Institute, Nanjing, China), according to the manufacturer’s instructions.

### Detection of ROS production

Intracellular superoxide anions were measured using the dihydroethidium (DHE) fluorescence probe and high performance liquid chromatography (HPLC) assay. The HUVECs were washed three times with DPBS before incubation with 10 μM DHE for 30 min at 37 °C. The nuclei were counterstained with DAPI (1 μg/mL). After washed with DPBS, the HUVECs were photographed using an inverted fluorescence microscopy (Olympus IX71, Japan). The ethidium (oxidized DHE) and DAPI fluorescence were quantified using Image-Pro Plus software (version 6.0, Media Cybernetics, MD, USA). ROS production was estimated from the ratio of ethidium/DAPI fluorescence.

### Statistical analysis

Statistical analyses were performed using GraphPad Prism 5 software (GraphPad Software, San Diego, CA, USA). Data are presented as the mean ± SEM. One-way analysis of variance (ANOVA) was used to determine the differences among treatment groups. Values of *P* < 0.05 were considered statistically significant.

## Results

### Identification of flavonoid monomers in the ethanolic extract

Since flavonoids are known to be potent radical scavengers, the extraction protocol was optimized to maximize the total flavonoid yield from *L. christinae* Hance. The plant species, source, and extraction conditions were strictly standardized to reduce the variability in phytochemical composition. Qualitative and quantitative analysis of flavonoid monomers in the crude ethanolic extract via HPLC revealed that rutin (3.36 mg/g), quercetin (0.83 mg/g), quercetin-3-methyl ether (0.17 mg/g), kaempferol (0.86 mg/g), isorhamnetin (0.35 mg/g), Isorhamnetin-robinobioside (4.11 mg/g), and chlorogenic acid (2.19 mg/g) could be used as markers for quality control and standardization.

### Total flavonoid, total phenolic, and total polysaccharide contents of the extract fractions

The total flavonoid (mg RE/g), total phenolic (mg GAE/g), and total polysaccharide (mg GE/g) contents of each fraction of *L. christinae* Hance extract are shown in Table 1. The flavonoid and phenolic contents were much higher in NB and EA fractions than in PE and AQ fractions (*P* < 0.05). The NB fraction also had the highest polysaccharide content among the four fractions.

**Table 1:** Amounts of total flavonoids, phenols and polysaccharides in each fraction isolated from ethanol extract of *Lysimachia christinae* Hance

	Total flavonoids (mg RE/g)	Total phenols (mg GAE/g)	Total polysaccharide (mg GE/g)
PE fraction	15.3 ± 1.56	15.6 ± 1.03	68.9 ± 4.75
EA fraction	37.5 ± 3.07	32.4 ± 3.08	85.4 ± 4.86
NB fraction	39.4 ± 4.55	41.1 ± 3.07	168.1 ± 7.07
AQ fraction	2.3 ± 0.12	29.1 ± 2.88	135.1 ± 6.29

Values are means±SEM of three determinations. *RE* rutin, *GAE* gallic acid, *GE* glucose, *PE* petroleum ether, *EA* ethyl acetate, *NB* n-butanol, *AQ* aqueous

### In vitro radical-scavenging activities of the extract fractions

DPPH, ABTS, superoxide anion, and hydroxyl radicals have been widely used for evaluating the antioxidant activity of chemical compounds, which is generally represented by IC_50_ (mg/mL), the concentration at which a compound shows 50% radical-scavenging activity. The IC_50_ values pertaining to the DPPH, ABTS, superoxide anion, and hydroxyl radical-scavenging activities of each extract fraction are indicated in Table 2. Collectively, the radical-scavenging activities of the four fractions decreased in the following order: NB fraction > EA fraction > PE fraction > AQ fraction. Thus, the NB fraction of the ethanolic extract of *L. christinae* Hance displayed the strongest radical-scavenging activity among the four fractions.

**Table 2:** In vitro radical-scavenging activities for each fraction isolated from ethanol extract of *Lysimachia christinae* Hance

Fraction/standard antioxidants	IC_50_ (mg/mL)			
	DPPH radical-scavenging activity	ABTS radical-scavenging activity	Superoxide anion radical-scavenging activity	Hydroxyl radical-scavenging activity
PE fraction	0.043	2.586	***	0.451
EA fraction	0.029	1.49	0.608	0.288
NB fraction	0.026	1.288	0.191	0.214
AQ fraction	0.056	3.712	0.801	0.763
BHT	0.023	–	–	–
TROLOX	–	1.471	–	–
VC	–	–	0.118	0.187

BHT, TROLOX and VC acted as positive control. ***: Cannot be detected;----: Not used as reference. *PE* petroleum ether, *EA* ethyl acetate, *NB* n-butanol, *AQ* aqueous, *DPPH* Diphenylpicrylhydrazyl, *ABTS* 2,2′-azino-bis(3- ethylbenzothiazoline-6-sulphonic acid)

### Protective effects of the extract fractions against H_2_O_2_-induced oxidative stress in HUVECs

**Fig. 1:**
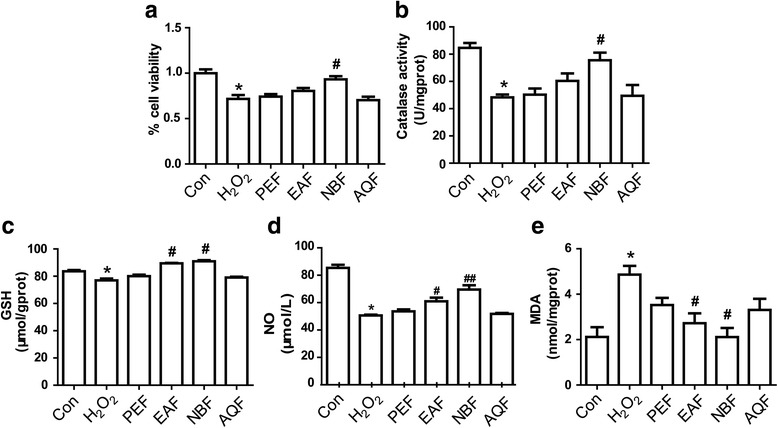
Effects of the four extract fractions on cell viability (**a**), catalase activity (**b**), and levels of glutathione (GSH) (**c**), Nitric Oxide(NO) (**d**) and malondialdehyde (MDA) (**e**) in human umbilical vein endothelial cells subjected to H_2_O_2_-induced oxidative stress. *P < 0.05 vs. control. ^#^*P* < 0.05 vs. H_2_O_2_ alone. *n* = 12. Con: control; PEF: petroleum ether fraction; EAF: ethyl acetate fraction; NBF: n-butanol fraction; AQF: aqueous fraction

The effects of the extract fractions on cell viability and oxidant-antioxidant balance were evaluated by estimating the catalase activity and levels of GSH, NO and MDA in HUVECs subjected to H_2_O_2_-induced oxidative stress. The cell viability, which was significantly reduced upon exposure to H_2_O_2_ (*P* < 0.05), was markedly increased by pretreatment with the NB fraction, whereas the EA, AQ, and PE fractions conferred lesser protection against H_2_O_2_-induced oxidative stress (Fig. 1a). Additionally, the NB fraction significantly improved the endogenous antioxidant activity and inhibited lipid peroxidation, as indicated by the increase in catalase activity and GSH, NO level (Fig. 1b–d) and decrease in MDA level (Fig. 1e) upon pretreatment with this fraction (*P* < 0.05 vs. H_2_O_2_ group).

### Effects of the four fractions on ROS production in HUVECs

**Fig. 2:**
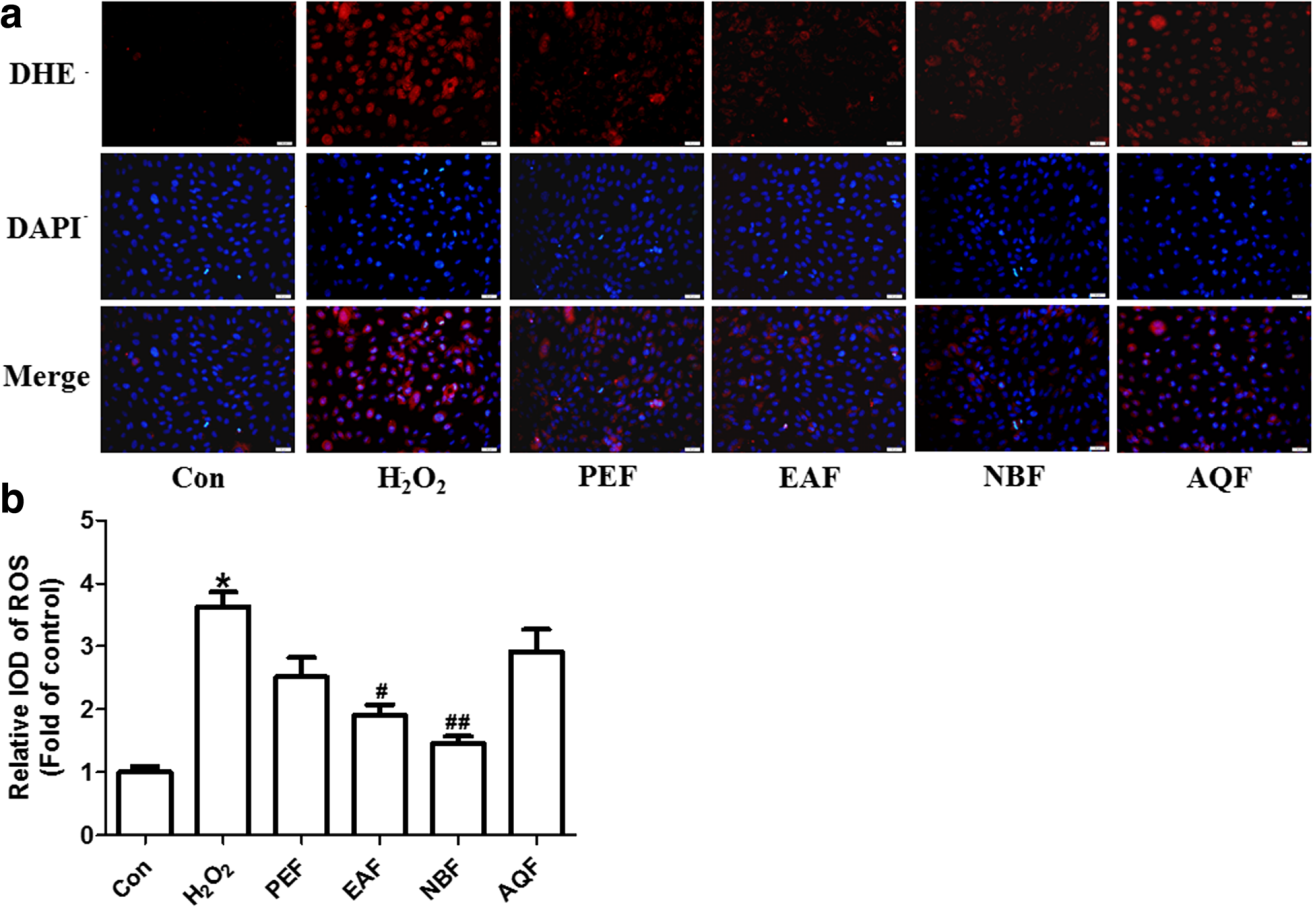
Effects of the four extract fractions on vascular reactive oxygen species (ROS) production in HUVECs. **a** The top panel shows representative images of DHE-stained cells, which fluoresce red owing to ROS-mediated oxidation of DHE to ethidium. The middle panel shows representative images of DAPI-stained nuclei that fluoresce blue. The bottom panel shows the merged fluorescence images (× 400 magnification). **b** Quantitative analysis of DHE fluorescence intensity. The average DHE fluorescence intensities were normalized to the fluorescence intensity of DAPI. All data are expressed as mean ± SEM. *n* = 6. *P < 0.05 vs. control. ^#^*P* < 0.05 and ^##^*P* < 0.01 vs. H_2_O_2_ alone. Con: control; PEF: petroleum ether fraction; EAF: ethyl acetate fraction; NBF: n-butanol fraction; AQF: aqueous fraction

The red fluorescence of ethidium in Fig. 2 indicates the presence of ROS in the HUVECs, and blue fluorescence indicates the nuclear stain DAPI. The ROS production, which was dramatically increased upon treatment with H_2_O_2_, was mildly inhibited by the four extract fractions in the order: NB fraction > EA fraction > PE fraction > AQ fraction (Fig. 2). Thus, the NB fraction was the most competent in preventing endothelial ROS accumulation, among the four fractions.

## Discussion

TCM has played a key role in protecting the health of Chinese people for over 1000 years. However, TCM formulations are generally complex mixtures composed of a variety of effective, ineffective, and even toxic ingredients. Therefore, it is essential to extract, separate, and purify the active constituents of TCM, in order to establish the intrinsic quality and improve the clinical efficacy of these medicines.

*L. christinae* Hance, a well-known TCM herb recorded in ancient literature, has been used to treat disease conditions such as stranguria, odynuria, brownish urine, jaundice, carbuncle, furuncle, and calculi [1, 2]. However, the active constituents responsible for these medicinal benefits have not yet been completely characterized. In the present study, the raw herb of *L. christinae* Hance was extracted with ethanol and separated into four fractions of different polarities. We then evaluated the endothelial protective effect and radical-scavenging activity of these fractions. Our results revealed that the NB fraction significantly reversed catalase activity and GSH, NO level reduced and MDA level increased by H_2_O_2_, suggesting that NB fraction could prevent H_2_O_2_-induced oxidative stress injury in HUVECs. To the best of our knowledge, this is the first study to investigate the active constituents and associated pharmacological activities of *L. christinae* Hance.

Several evidences suggest that oxidative stress plays a central role in the pathogenesis of cardiovascular diseases. Antioxidants can delay or inhibit cellular oxidative damage by blocking the initiation or propagation of the oxidative chain reaction. Thus, the use of antioxidants and free radical scavengers has been accepted as an important strategy for the prevention or treatment of various cardiovascular diseases [16]. In this study, we evaluated the radical-scavenging activities of the four extract fractions using in vitro oxidative injury models of endothelium. Our results demonstrated that the NB fraction showed the strongest radical-scavenging and antioxidant activities in the endothelium and thus the most potent endothelial protective effect among the four fractions.

The observed differences in the radical-scavenging and antioxidant activities of the four ethanolic extract fractions may be attributed to differences in their active constituents. Phenols, the most abundant antioxidants among plant secondary metabolites, have shown promising antioxidant activity both in vivo and in vitro [6]. Phenolic compounds generally contain one or more aromatic rings with one or more hydroxyl groups, and their antioxidant activity increases with an increase in the number of free hydroxyl groups and conjugation of side chains to the aromatic rings [17]. The antioxidant properties of flavonoids, a major group of polyphenols derived from 2-phenylchromone and comprising more than 10,000 compounds, have also been extensively investigated over the years [18]. In the present study, the total phenolic and total flavonoid contents were much higher in the NB fraction than in other fractions, which could well explain its higher antioxidant and radical-scavenging activities. However, the polysaccharide content of the four fractions decreased in the following order: NB fraction > AQ fraction > EA fraction > PE fraction (Table 1), which did not coincide with the orders of their radical-scavenging (Table 2) and antioxidant activities (Fig. 1b–d). Thus, the phenolic and flavonoid components of the extract fractions may be chiefly responsible for their antioxidant properties [19, 20].

## Conclusions

In the present study, we separated the crude ethanolic extract of *L. christinae* Hance into four fractions of different polarities and demonstrated that the NB fraction showed the most potent antioxidant and endothelial protective effects, which may be attributed to its high phenolic and flavonoid contents. Our work has thus attempted to characterize the active ingredients and associated pharmacological activities of *L. christinae* Hance*,* which may aid in improving its application for disease prevention and treatment. Future research should focus on the isolation, purification, and characterization of the bioactive constituents in the NB fraction of the ethanolic extract.

## Abbreviations

H_2_O_2_: Hydrogen Peroxide
HPLC: High performance liquid chromatography
HUVECs: Human umbilical vein endothelial cells
IC_50_: The concentration with 50% scavenging activity
ROS: Reactive oxygen species
